# A Glider-Assisted Link Disruption Restoration Mechanism in Underwater Acoustic Sensor Networks

**DOI:** 10.3390/s18020501

**Published:** 2018-02-07

**Authors:** Zhigang Jin, Ning Wang, Yishan Su, Qiuling Yang

**Affiliations:** 1 School of Electrical and Information Engineering, Tianjin University, Tianjin 300072, China; cathy201233@tju.edu.cn; 2 College of Information Science and Technology, Hainan University, Haikou 570228, China; yql0515@163.com

**Keywords:** underwater acoustics sensor networks, glider, sawtooth motion, link-disruption, restoration, network connectivity

## Abstract

Underwater acoustic sensor networks (UASNs) have become a hot research topic. In UASNs, nodes can be affected by ocean currents and external forces, which could result in sudden link disruption. Therefore, designing a flexible and efficient link disruption restoration mechanism to ensure the network connectivity is a challenge. In the paper, we propose a glider-assisted restoration mechanism which includes link disruption recognition and related link restoring mechanism. In the link disruption recognition mechanism, the cluster heads collect the link disruption information and then schedule gliders acting as relay nodes to restore the disrupted link. Considering the glider’s sawtooth motion, we design a relay location optimization algorithm with a consideration of both the glider’s trajectory and acoustic channel attenuation model. The utility function is established by minimizing the channel attenuation and the optimal location of glider is solved by a multiplier method. The glider-assisted restoration mechanism can greatly improve the packet delivery rate and reduce the communication energy consumption and it is more general for the restoration of different link disruption scenarios. The simulation results show that glider-assisted restoration mechanism can improve the delivery rate of data packets by 15–33% compared with cooperative opportunistic routing (OVAR), the hop-by-hop vector-based forwarding (HH-VBF) and the vector based forward (VBF) methods, and reduce communication energy consumption by 20–58% for a typical network’s setting.

## 1. Introduction

For decades, marine technology has attracted a lot of interest. In the marine technology field, underwater acoustic sensor networks (UASNs) have made significant achievements in many aspects of underwater environmental exploration, such as ocean data collection, monitoring of water pollution, underwater disaster warning [1], and so on. Therefore, the study on UASNs has received increasing attention.

However, due to the harsh communication environment, acoustic communication among nodes in UASNs has much more difficulties than that in terrestrial sensor networks. The first one is that the communication among underwater sensor nodes must rely on acoustic channels instead of radio channels for long-range communications. That is because radio signals undergo rapid attenuation and that makes the communication range decrease sharply in UASNs. For example, the reported transmission range of Berkeley Mica 2 Motes is 120 cm at 433 MHz [2]. Therefore, the acoustic signals have become the typical signal carrier due to their relatively lower attenuation. Many studies have been done on the realization of underwater communications. The method proposed by Won and Park in [3] shows the design and implementation of an omni-directional underwater acoustic micro-modem and it provides the development of the cylindrically shaped micro-modem guaranteeing an omni-directional beam pattern in three dimensions [4].

However, the fact that the speed of acoustic signals is five-fold slower than the radio wave makes the end-to-end delay longer. The second one is that many factors make acoustic links among the sensor nodes dynamically changed with the time flow. One of the factor is that the location of a node tends to change over time due to water currents, drift and contact with marine organisms. Another factor is that the speed of sound is influenced by salinity, density and temperature of the water medium, and these factors are heterogeneous, which makes the channels between nodes vary with time. In the sea, those parameters present characteristics of stratification and thus the speed of sound is also stratified and varies with the depth. These factors have led to new studies focused on the optimization of mobility, coverage and connectivity [5]. Even if networks meet the connectivity conditions at the time of initial deployment, the communication could suffer a sudden disruption among nodes due to the time-varying acoustic communicating links. Furthermore, the sudden disruption of links can lead to the fact that the overall network connectivity and reliability might not be able to meet the requirements of application. All of these characteristics make the communication quality of UASNs unstable and the design of stable UASNs very tough. Therefore, in order to improve the connectivity and reliability of networks, it is important to design a mechanism to flexibly realize link disruption restoration over time.

There is the problem that acoustic channels are time-varying, which can cause the links among nodes to be disrupted in UASNs. To solve this problem, many routing algorithms and topology maintenance mechanisms are proposed. The hole avoidance algorithm in [6] pays attention to energy hole and coverage hole avoidance. It can take advantage of redundant overlapping and repair a coverage hole during network operation with the least number of nodes. However, the coverage does not necessarily lead to network connectivity. The reliable and stable communication links also affect the network connectivity and thus affect the quality of entire network. In [7], a distributed reactive shadow zone and delay-aware routing protocol (SZODAR) for UASNs is proposed. It plays a certain role in maintaining the connectivity and reliability of networks. However, like SZODAR, many methods just rely on installing additional autonomously moveable power devices in common sensor nodes to move nodes up and down and achieve the desired effect. That is to say, these methods require more energy consumption when the nodes are adjusted up and down. However, as we all know that the energy of underwater nodes is extremely limited because the nodes are powered by batteries. Furthermore, nodes are anchored to the sea bottom in most cases so that it is difficult to recharge the batteries. Therefore, to some extent, these methods will shorten the lifetime of nodes and make networks disconnected and unreliable faster.

Moreover, the above algorithms do not pay attention to using advanced nodes that can be free to move to maintain the connectivity of networks. Considering that many data acquisition tasks in UASNs are completed by both static nodes and autonomous mobile nodes [8,9], we apply autonomous mobile nodes that perform data acquisition tasks to assist in repairing the link-disruption to maintain network topology more flexibly and efficiently. As a new type of monitoring device with unique driving mode, gliders have advantages of lower energy consumption, longer run-time, lower noise and lower energy consumption compared with AUVs [9]. Therefore, gliders are usually used in collecting environmental parameters missions in many long-time and large-scale three-dimensional ocean [10,11]. Therefore, for less real-time networks, underwater gliders can assist the network in restoring a link disruption while carrying out data acquisition tasks. However, due to their single sawtooth trajectory on the vertical plane, it is necessary to fully consider the movement characteristics of gliders when we design an underwater acoustic channel link restoration mechanism. It is also of great significance for maintaining network topology and restoring the connectivity and reliability in UASNs.

In view of above problems, we propose a link disruption recognition and glider-scheduling algorithm (LDR-GS) and a glider-assisted relay location optimization algorithm (GALO) to assist in restoring link disruptions using existing nearby gliders. In LDR-GS, the cluster head nodes collect the link state and schedule gliders to prepare restoring the link-disruption. Since the location of restoring the link-disruption is limited due to the unique trajectory of gliders, GALO is proposed to select the optimal position for a glider as the relay node of the disrupted link, to accurately restore the connectivity and reliability of network. In GALO, the optimal location is selected by combining the unique characteristics of underwater gliders and the channel attenuation model. Combining LDR-GS and GALO, connectivity of the networks can be restored.

The rest of this paper is organized as follows: in Section 2, related works on mechanisms for restoring underwater network connectivity are discussed briefly. In Section 3, the proposed network model and LDR-GS mechanism are introduced. In Section 4, GALO algorithm is described in detail. The simulation results are shown and discussed in Section 5. At last, we conclude this paper in Section 6.

## 2. Related Work 

The technology to improve connectivity and reliability of sensor networks is a hot topic nowadays. There are several kinds of methods aiming to improve connectivity and reliability to meet the requirement of applications, such as the algorithms for the routing layer and MAC layer [12,13,14,15,16], the nodes complement about dynamic nodes and static nodes [17,18,19] and so on. In this section, we provide a review on research works on this topic.

Communication link restoration and network topology maintenance problems exist in a variety of network designs from the viewpoint of routing and the MAC layer. A centralized (C-TCSIC) and a distributed topology control algorithm (D-TCSIC) are proposed in [12]. C-TCSIC and D-TCSIC integrate power control with channel allocation to create a bidirectional and a collision-free channel connection in cognitive radio networks (CRNs). Theoretical analysis shows that the algorithm can guarantee two-channel connectivity without conflicts. However, this algorithm cannot effectively complete the restoration of link disruption among nodes for the complex environment in UASNs. Some problems arise if the transmission power is only adjusted according to the communication channel condition among adjacent nodes to restore the link disruption. This is because the disruption of communication in two nodes is not only caused by the increase of communication distance, so merely increasing the transmission power not only causes a large energy overhead but also easily causes interference to other nodes and reduces the channel capacity. For sparse networks, the authors in [16] propose a network analytic model and designed an any-to-any communication scheme. The model considers the problem of real-time message-delivery which is solved by integer linear programming and heuristics. The model proposed in [16] can reduce the complexity and improve the efficiency of networks and it is helpful for network simulation. With distributed beacons, the authors in [13] propose a novel cooperative opportunistic routing (OVAR) scheme for UASNs and build a contiguity graph at each hop and select a forwarding set that maintains the best balance between reliability and energy efficiency. OVAR can also choose a forwarding set from any direction of the sender, which increases its flexibility to bypass any type of hole and has the least deviation from the best path.

In addition to the research on such routing and MAC layers, there are many related studies on network topology optimization from the perspective of node deployment [8,20,21,22]. Node deployment can be divided into static deployment and dynamic deployment. Static deployment consists of random deployment and deterministic deployment. However, whether it is random deployment or deterministic deployment, underwater acoustic channels will dynamically change with the water environment, resulting in disrupted links and network partitioning. In [17], aiming at the problem of link disruption in sensor networks, the network performance is restored by sowing second-generation nodes. Although some positive results are obtained, considering the uncertainty of node spreading, a larger node redundancy will result. A two-node deployment method is proposed in [18] for network partitioning phenomenon in multi-hop UASNs, which repairs the disrupted links by adjusting the depth of nodes. This method does not produce more node redundancy like that in [17]. However, it requires nodes to automatically adjust their depth, which requires much more flexibility from nodes. Similarly, Ref.[19] makes use of the virtual force algorithm to make the nodes cover the network connection holes. The algorithm can reduce the node redundancy, but requires a higher computational capability and self-mobility capability and the driving force of node movement also generates a large energy expenditure. However, the method has a great limitation for underwater sensor nodes due to the difficulty of charging and replacing the battery of common sensor nodes.

In order to improve the flexibility and generality of network of link disruption restoration and save the energy of common sensor nodes to reinforce the connectivity of the network, many researchers have begun to study the use of movable and rechargeable underwater vehicles to assist in underwater monitoring tasks. Based on the motion characteristics of underwater vehicles, Ref.[9,23] have made a related study on the location prediction of dynamic nodes and the communication among nodes. The authors in [24] propose an AUV-aided underwater routing protocol (AURP) for UASNs. AURP uses AUV as a relay node to assist common sensor nodes collecting information and gives full play to the initiative of the mobile node and reduces the load of the network static nodes. Learn from this idea, taking into account the strong initiative and the low energy consumption of gliders, we propose a glider-assisted link-disruption restoration mechanism to restore interrupted links in UASNs.

## 3. The Glider-Assisted Link-Disruption Restoration Mechanism

We first describe the network model that is the basis for our mechanism. Due to the wide range of deployment in wireless sensor networks and the complex communication environment, the network structure is generally clustered in UASNs. After that, we introduce the process of the glider-assisted link disruption restoration mechanism. Next, we design a reasonable link disruption recognition and glider-scheduling (LDR-GS) mechanism as the first part of the restoration. In LDR-GS every cluster head node can learn about the link interruption and transfer the status information to the glider when the glider is periodically close to the cluster head node. The previous work can fully prepare for glider-assisted link restoration.

### 3.1. The Network Model

In the water volume D to be monitored, the glider-assisted link disruption restoration problem considered in this paper is based on the pre-existing hybrid deployment of static and dynamic nodes. Assume that the original sensor nodes deployment has satisfied the coverage and connectivity conditions.

**Figure 1:**
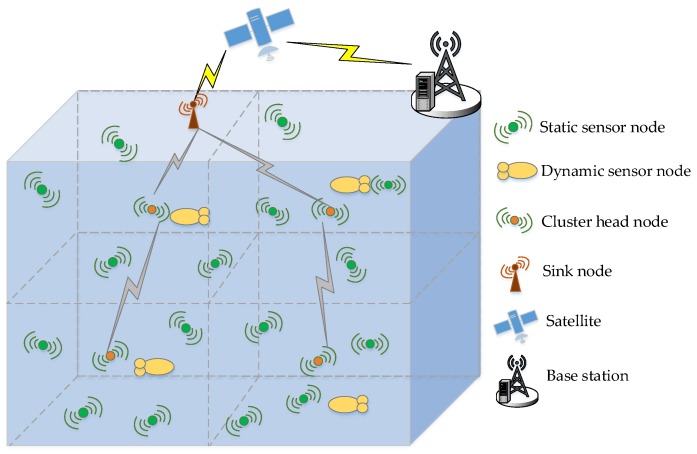
Network model.

As shown in Figure 1, the entire volume is divided into several sub-volumes and the network is a clustered structure. Each sub-volume contains three kinds of nodes including a cluster head node *h_i_*, a glider *g_i_* and multiple static sensor nodes $S_{i}=\left\{s_{i}^{1},s_{i}^{2},\ldots ,s_{i}^{j}\right\}$. A static node is anchored to the sea floor by cables and floats in the water aided by buoyancy devices. Its function is to collect and forward the environmental data to complete the conventional monitoring tasks. All static nodes have the same sensing range and communication range. The cluster head node is the only one in each sub-volume. The cluster head node is responsible for receiving the data forwarded by the static and dynamic nodes. In addition to this, the cluster head node can collect the status information of whether the link is disrupted in its sub-volume. Every cluster head node then sends the collected information to the cluster head nodes of the upper sub-volume so that the collected data is transmitted to sink nodes layer by layer. Finally, the sink node will send the combined data to a satellite or shore base station through radio communication. The dynamic node in our paper is a glider. On the one hand, the glider has a common function with static nodes. It can collect and forward environmental data. On the other hand, to a certain extent the dynamic nodes can freely move and act as an advanced sensor node. Every glider would periodically pass through the cluster head nodes to forward the collected data information to the cluster head node in its sub-volume.

Although the static nodes in the network are fixed to the bottom by cables, the location of the static nodes will be disturbed due to the external forces such as water currents and contact with marine animals. Compared with the nodes that are not fixed, the anchoring nodes are relatively less affected by the water flow. Their position change volume is in a spherical coronal surface, which also returns to the original position as the water flow changes over time. The change of the node position will directly affect the quality of underwater acoustic links. In addition, the quality of the underwater acoustic links will also be affected by the sea surface fluctuation. Therefore, different sea surface fluctuations may cause different signal propagation paths. These time-varying factors will temporarily reduce the quality of underwater acoustic links. In severe cases, the communication link will be disrupted and the network connectivity will be degraded. If the network just waits for the environment to return to its original state, it will inevitably cause link disruption for a longer time, resulting in longer transmission delays and greater waste of network resources caused by data retransmission.

In addition to the problem of link disruption caused by the change of underwater acoustic channel, there are several other reasons why the acoustic links can be disrupted in UASNs. These problems include the failure of nodes, the changed direction of hydrophones due to currents and so on. In this paper, we only cover the link disruption during the networking process of sensor nodes, that is, we do not consider the case that underwater node is disrupted. We assume that the sensor nodes in our network model carry omni-directional hydrophones and we do not consider the change of hydrophone direction.

### 3.2. The Glider-Assisted Link Disruption Restoration Process

In view of the above analysis, it is necessary to design a mechanism to restore the link disruption. We first propose the link disruption recognition and glider-scheduling (LDR-GS) mechanism for identifying the disrupted links and collecting the link disruption information in preparation for scheduling glider for restoring the disrupted link. The glider reaches the location of a disrupted link to complete the restoration of the link disruption to restore the network connectivity. In the next section we design GALO to find out the best location to repair the link more accurately due to the unique trajectory of the glider, but in this part we just introduce the LDR-GS mechanism.

**Figure 2:**
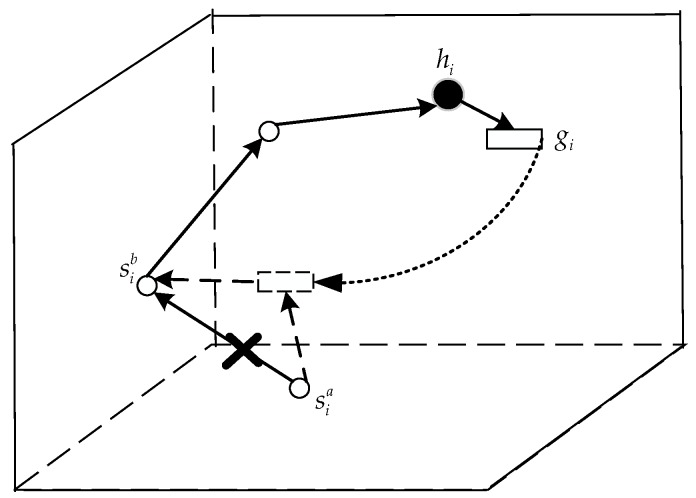
The process of glider-assisted link-disruption restoration.

The cluster head node *hi* in the sub-volume *i* is responsible for collecting and forwarding the data packets sent by all the cluster members *S_i_* in the cluster and the glider *g_i_*, and finally sends the data to the surface sink node. Supposing that the routing table of the cluster head holds all the routing information in the volume, and the route arrived by each sensor node is routed according to the shortest path, that is, the path reached by each node is unique. In this network, underwater nodes automatically go to sleep from the active state when no data needs to be received or sent. While the node is sleeping, it no longer sends data packets. When the state changes, the node sends the data packet to update the status information. Glider *g_i_* in this volume periodically approaches the head nodes *h_i_* and the collected environmental data is forwarded to *h_i_*. At the same time *h_i_* forwards the link disruption information of cluster members to *g_i_* so that the glider *g_i_* can repair the link disruption is a timely way as the relay node. A schematic diagram of this is shown in Figure 2.

**Figure 3:**
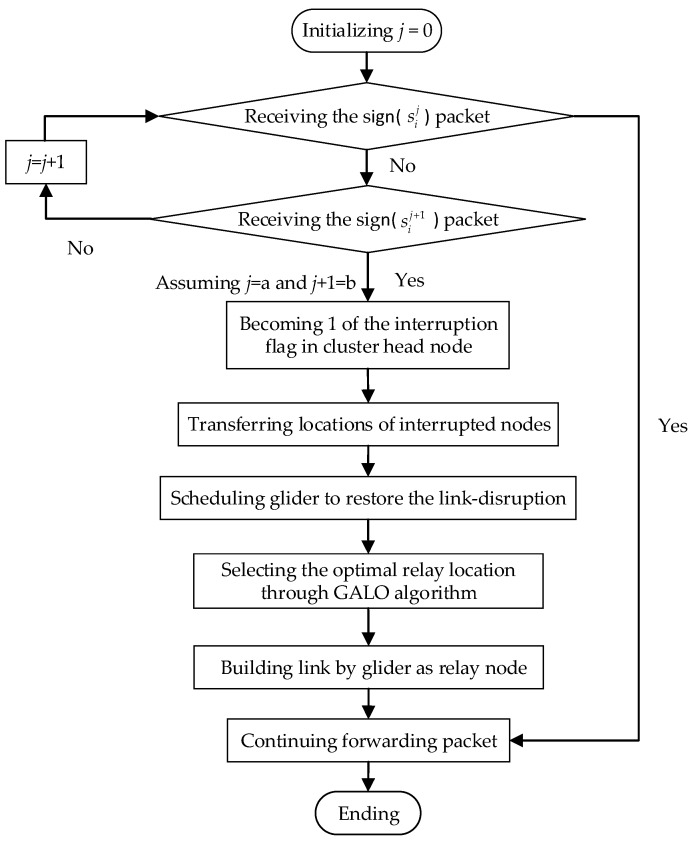
The overall process of glider-assisted link disruption restoration mechanism.

Therefore, in order to get the state information of cluster members for cluster head nodes in time, cluster members need to periodically report their current location and current status to cluster heads through the LDR-GS mechanism and it is necessary to accurately schedule the glider for link healing when the cluster head node finds any link disruption through the GALO algorithm. The process is shown in Figure 3.

### 3.3. The LDR-GS Mechanism

The details of LDR-GS mechanism follows the steps below:

Step 1: The static sensor node $s_{i}^{j}$ sends data packet sign ($s_{i}^{j}$), which contains the node’s own position information, to its cluster head node *h_i_* every time period.
Step 2: If the cluster head *h_i_* receives the sign ($s_{i}^{j}$) packet sent by $s_{i}^{j}$, it indicates that there is no disruption of all links between static node $s_{i}^{j}$ and the cluster head *h_i_*. If the cluster head *h_i_* does not receive the sign ($s_{i}^{j}$) packet sent by $s_{i}^{j}$ but receives the sign ($s_{i}^{j+1}$) packet of the next hop $s_{i}^{j+1}$, then it judges the link *e_j,j+_*_1_ between $s_{i}^{j}$ and $s_{i}^{j+1}$ to be disrupted and the link disruption identifier in *h_i_* therein becomes *δ_j,j_*_+1_ = 1. If the cluster head *h_i_* does not receive the sign ($s_{i}^{j+1}$) packet sent by $s_{i}^{j=+1}$, then it does *j* = *j* + 1 and continues to judge until the cluster head can receive from the next hop. Then the cluster head can judge the disrupted link *e_ab_*, where *b* = *a* + 1. If there are some simultaneous link disruptions, the glider will prioritize the link closest to the cluster head node to ensure the connectivity of more nodes.
Step 3: If the link disruption flag *δ_ab_* is 0, the glider completes the data forwarding and continues to collect data according to the original running track. If there is a link-disruption, that is the link disruption flag *δ_ab_* is not all 0, *h_i_* sends a repair (*e_ab_*) packet containing the location information of the two nodes to *g_i_* when *g_i_* periodically approaches *h_i_* for data forwarding, and then starts scheduling the glider to repair the link disruption.
Step 4: If *g_i_* receives the repair (*e_ab_*) packet, the glider’s link interrupt flag becomes *δ_ab_* = 1. The disrupted link is repaired by selecting the appropriate trajectory according to the motion characteristics of the glider *g_i_*.
Step 5: After *g_i_* completes the link-disruption restoring command issued by *h_i_*, the glider’s link interruption flag *δ_ab_* becomes 0, indicating that the link *e_ab_* repair task delivered by the cluster head has been completed. In this case, *g_i_* returns to the original position again and sets the link disruption flag of *h_i_* to 0, and continues the environmental monitoring task.

## 4. The GALO Algorithm

It is necessary to design a glider-assisted relay location optimization algorithm (GALO) so that the glider can calculate the optimal relay location and complete the link restoring when the glider receives the repair command (*e_ab_*) issued by a cluster head. In this section, we first describe the problem and the process of establishing optimization model. Since the glider has a special sawtooth movement, we model it based on its kinematics to solve the problem. Then we briefly introduce the motion model of the “Petrel” glider as the basis for model building. In addition, we use the position with the smallest signal attenuation as the optimization target. Due to the special trajectory of gliders, the glider probably cannot arrive the calculated optimal position and therefore depending on the different cases we design two solutions for the glider’s trajectory.

### 4.1. The Problem Description

In GALO, according to the received location of the link disruption and the position of the glider itself, the optimal relay location is calculated based on the glider’s motion characteristics. The schematic diagram of GALO is shown in Figure 3. Assume that both node $s_{i}^{a}$ and node $s_{i}^{b}$ are undamaged, that is, their data sensing, data sending and receiving functions work normally. The disruption of the underwater acoustic channel between two nodes is then only a temporary suspension of the communication link due to changes in the water environment. Therefore, the glider is needed to repair the link between these two nodes. At this time, the glider serves as a relay node between two nodes to repair the damaged link and transmits the data back to the cluster head node for data fusion. Then the cluster head node transmits the message to the water surface aggregation node. In this problem, we need to calculate the best glider trajectory based on the glider’s motion characteristics. We treat this problem as a Nonlinear Program (NLP) problem. In this NLP problem, the position selection of the glider satisfies the signal-noise-ratio threshold of the receiver. Combining with the glider’s motion characteristics, we minimize the signal loss of $s_{i}^{a}-g_{i}$ and $g_{i}-s_{i}^{b}$ through selecting the optimal position as the objective to establish multi-objective function:

(1) $$\min\left\{TL_{ag},TL_{gb}\right\}$$

When making decision in the game theory, the problem of multi-objective optimization is often solved by ideal point method. That is to say, the minimum value point of each component objective function is calculated as the ideal value of the component objective function. Then, the optimal solution of the original multi-objective programming problem is obtained by the way that the multi-objective function approximates the corresponding ideal value as much as possible in the feasible domain. The evaluation function is:

(2) $$u(f(x))=\left|TL_{ag}-T{L_{ag}}^{*}\right|+\left|TL_{gb}-T{L_{gb}}^{*}\right|$$

(3) $$TL_{ag}=\chi \log r_{ag}+\alpha r_{ag}\cdot 10^{-3}$$

(4) $$TL_{gb}=\chi \log r_{gb}+\alpha r_{gb}\cdot 10^{-3}$$

where $r_{ag}=∥x-y∥$, $r_{gb}=∥x-z∥$, $x={\left(x_{1},x_{2},x_{3}\right)}^{T}$ is the coordinate vector of the glider, $y={\left(y_{1},y_{2},y_{3}\right)}^{T}$ is the coordinate vector of the sensor node $s_{i}^{a}$, and $z={\left(z_{1},z_{2},z_{3}\right)}^{T}$ is the coordinate vector of the sensor node $s_{i}^{b}$. *χ* is a parameter used to calculate the extended loss caused by the continuous expansion of the wavefront during the propagation of the sound wave. For the shallow sea, the wavefront expands according to the law of the cylinder side and at this time *χ* = 10. The wavefront expands spherically for the deep sea and at this time *χ* = 20. *α* is used to calculate the absorption coefficient of sound attenuation due to medium viscosity and heat conduction. The corresponding empirical formula is $\alpha =\frac{0.1f}{1+f^{2}}+\frac{40f}{4100+f^{2}}+2.75*10^{-4}f^{2}+0.003$. Therefore, the NLP problem can be expressed using Equation (5):

(5) $$\begin{array}{l} \min\left\{\left|TL_{ag}-T{L_{ag}}^{*}\right|+\left|TL_{gb}-T{L_{gb}}^{*}\right|\right\}\\ s.t.\\ C1.\;TL_{ag}<TL_{th1}\\ C2.\;TL_{gb}<TL_{th2}\\ C3.\;c_{s}(x),c_{t}(x)\in trail(x) \end{array}$$

In order to ensure the signal quality of receiver, it is necessary to establish Equation (6), where *SL* (Unit: dB) is the signal level sent by the source node. *TL* is the transmission loss. *NL* is the noise level and *DL* is the directional gain:

(6) $$SL-TL-NL+DL\geq Th_{receiver}$$

Therefore, this condition needs to be established as *TL* ≤ *SL* − *NL* + *DL* − *Th_receiver_*. The constraint condition *C*1 indicates that the transmission loss from $s_{i}^{a}$ to *g_i_* should be lower than a loss threshold *TL_th_*_1_. The constraint condition *C*2 indicates that the transmission loss from *g_i_* to $s_{i}^{b}$ should be lower than the loss threshold *TL_th_*_2_. The formula in the constraint *C*3 indicates the movement trajectory of the glider should be considered when the link is repaired, and the starting and ending positions are certain points on the movement trajectory. In other words, different trajectories result in different feasible regions of the objective function.

### 4.2. The Glider Movement Description

To meet the constraints in *C*3, the trajectory of glider *g_i_* also needs to be studied. Taking the “Petrel” glider as an example, the motion characteristics of the glider are studied as follows: in its gliding mode, the glider follows a sawtooth profile through the buoyancy adjusting unit and the attitude adjusting unit. The spiral gyratory movement is achieved by combining the sawtooth movement with the roll adjustment unit.

It is necessary to consider the stress conditions of the glider to obtain the kinematics formula. Specifically, based on the kinetics formula, the kinematic parameters of the glider at any moment are solved by the Runge-Kutta method with variable step size. Due to the relatively higher efficiency of the glider’s buoyancy control unit, the sawtooth profile motion controlled by the buoyancy control unit and the attitude adjustment unit is more suitable for long-range monitoring tasks. The adjustment of the control parameters can control the movement data of the “Petrel” and the relationship among these speed parameters is shown in Equation (7):

(7) $$\{\begin{array}{l} V_{x}=u\cos\theta \cos\psi +v\sin\psi \sin\phi -v\sin\theta \cos\psi \cos\phi +w\sin\psi \cos\phi +w\sin\theta \cos\psi \sin\phi \\ V_{y}=u\sin\theta +v\cos\theta \cos\phi -w\cos\theta \sin\phi \\ V_{z}=-u\cos\theta \sin\psi +v\cos\psi \sin\phi +v\sin\theta \sin\psi \cos\phi +w\cos\psi \cos\phi -w\sin\theta \sin\psi \sin\phi \end{array}$$

where (*V_x_*,*V_y_*,*V_z_*) is the speed vector of the underwater glider floating in the ground coordinate system and (*u*,*v*,*w*) is the coordinates of the velocity vector in the body at the center of the float. *φ*, *ψ* and *θ* are the roll angle, pitch angle and flight angle, respectively. From the velocity vector formula of the above equation the underwater glider trajectory can be obtained. The trajectory *trail*(*x*) of the underwater glider is the integral of the speed vector.

### 4.3. The GALO Algorithm

As the pitch angle θ of the glider during gliding is related to the system dynamics parameters, under the control parameters of the “Petrel”, the range of the pitch angles of the glider in steady-state gliding is (−69.5°,−9.2°)∪(9.2°,69.5°). Therefore, due to the limitation of the movement characteristics, there are two situations for solving the nonlinear programming problem:

Case 1: The glider’s trajectory intersects with the volume where the objective function is optimal.
Case 2: The glider’s trajectory does not intersect with the volume where the objective function is optimal.

For different situations, we need to have different methods. For Case 1 where the trajectory intersects with the optimal region, the optimal solution of the problem can be obtained for the problem. Then the NLP problem can be solved by the multiplier method to obtain the optimal solution to the problem. For Case 2 where the trajectory does not intersect with the optimal region, the optimal solution to the problem does not exist. Then the problem can be transformed into solving the minimum distance between the glider trajectory coverage volume *A*_1_ and the optimal region *A*_2_. We take this point that meets the minimum distance on the trajectory as the suboptimal solution to the NLP problem. That is, the problem is transformed into the objective function min{*d*(*A*_1_,*A*_2_)}. Specific solution steps are as follows:

Case 1: The glider’s trajectory intersects with the volume where the objective function is optimal. The problem can be solved by multiplier method (PHP) and the corresponding augmented Lagrange function is shown as (8):
  (8) $$\begin{array}{l} M(x,\lambda ,\delta )=\sqrt{{\left(TL_{ag}-T{L_{ag}}^{*}\right)}^{2}+{\left(TL_{gb}-T{L_{gb}}^{*}\right)}^{2}}-\lambda_{3}\cdot trail(x)+\frac{\delta }{2}trail^{2}(x)+\frac{1}{2\delta }\{[\max\{0,\lambda_{1}-\delta (TL_{th1}\\ -TL_{ag})\}{]}^{2}-{\lambda_{1}}^{2}\}+\frac{1}{2\delta }\{{\left[\max\{0,\lambda_{2}-\delta (TL_{th2}-TL_{gb})\}\right]}^{2}-{\lambda_{2}}^{2}\} \end{array}$$
  where *λ* = (*λ*_1_,*λ*_2_,*λ*_3_)^T^ is the corresponding Lagrange multiplier vector. The corresponding multiplier iteration formula is shown as (9):
  (9) $$\begin{array}{l} {\lambda_{1}}^{k+1}={\lambda_{1}}^{k}-\delta (TL_{th1}-TL_{ag})\\ {\lambda_{2}}^{k+1}={\lambda_{2}}^{k}-\delta (TL_{th2}-TL_{gb})\\ {\lambda_{3}}^{k+1}=\max\{0,{\lambda_{3}}^{k}-\delta trail(x^{k})\} \end{array}$$
  The original problem (5) is converted to the following unconstrained optimization problem (10):
  (10) $$\min\{M(x,\lambda^{k},\delta_{k})\}$$
  By separately taking partial derivative of the variable *x*_1_,*x*_2_,*x*_3_ and making $\frac{\partial M}{\partial x_{1}}=\frac{\partial M}{\partial x_{2}}=\frac{\partial M}{\partial x_{3}}=0$, the optimal solution $x^{k}={\left({x_{1}}^{k},{x_{2}}^{k},{x_{3}}^{k}\right)}^{T}$ can be obtained separately and then substituted into Equation (11) to calculate whether the termination rule is established:
  (11) $$\phi_{k}:={\left\{trail^{2}(x)+{\left[\min\{TH_{th1}(x)-TL_{ag}(x),\frac{{\lambda_{1}}^{k}}{\delta }\}\right]}^{2}+{\left[\min\{TH_{th2}(x)-TL_{gb}(x),\frac{{\lambda_{2}}^{k}}{\delta }\}\right]}^{2}\right\}}^{\frac{1}{2}}<\varepsilon$$
  If Equation (11) works, the solution is the approximate optimal solution, and if not, it will be brought into Equation (9) to continue calculating for the next iteration until Equation (11) works. At this point, the optimal relay position of glider for link restoration is $x^{*}={\left({x_{1}}^{*},{x_{2}}^{*},{x_{3}}^{*}\right)}^{T}$.
Case 2: The glider’s trajectory does not intersect with the volume where the objective function is optimal. Since the glider’s trajectory is limited, the glider may not be able to go through the optimal position represented by Equation (5). The nearest point in the volume *A*_1_ to the volume *A*_2_ is selected as the sub-optimal solution. Volume *A*_1_ is the glider’s trajectory coverage volume and volume *A*_2_ is the zone defined by the *C*1 and *C*2 constraints. That is, the range indicated by the volume *A*_1_ is expressed by Equation (12), and the range indicated by the volume *A*_2_ is expressed by Equation (13):
  (12) trail(x)=0
  (13) $$\begin{array}{l} \min\left\{\sqrt{{\left(TL_{ag}-T{L_{ag}}^{*}\right)}^{2}+{\left(TL_{gb}-T{L_{gb}}^{*}\right)}^{2}}\right\}\\ s.t.\\ C1.\;TL_{ag}<TL_{th1}\\ C2.\;TL_{gb}<TL_{th2} \end{array}$$
  The original problem of Equation (5) is thus transformed into a new problem for solving the suboptimal solution, as shown in Equation (14). The solution obtained to achieve the shortest distance between the two regions is the sub-optimal solution of the original problem. It is the position for the glider to restore the link:
  (14) $$\min\{d(A_{1},A_{2})\}$$

Based on the above calculation, the glider can arrive the optimal or sub-optimal position to restore the link disruption as a relay node. However, if the data packet needs a long time to be transmitted or the link needs a long time to be restored, there may be a link disruption between the node and the glider due to currents. Thence designing a method to withstand currents is necessary when the glider prepares restoring the disrupted link. We consider that the glider has two kinds of movement (sawtooth and spiral movement). It achieves a sawtooth profile through the buoyancy adjusting unit and the attitude adjusting unit. The spiral movement is achieved by combining the sawtooth movement with the roll adjustment unit. When the glider arrives at the calculated position, it transforms its movement mode into a spiral movement. That is because it can move within a relatively small volume in a spiral movement. In additions, the maximum total speed, vertical speed and horizontal speed of the glider are 2.05 m/s, 1.88 m/s and 1.93 m/s, respectively. As an important performance parameter of counteracting currents, the greater horizontal speed indicates a stronger anti-flow ability [10,11], and higher reliability in the harsh environment. In addition, the communication between node and glider with a specific trajectory has been studied [9]. Therefore, the conversion of movement model can complete the communication between node and glider without disruption.

In this networking process, the cluster head node can calculate the probability of the link disruption in this cluster. In general, the probability of link disruption can be calculated as $F_{ab}=Pr(log_{2}(1+{\left|H_{ab}\right|}^{2}SNR_{ab})<R_{A})=Pr(\left|H_{ab}\right|<\frac{2^{R_{A}}-1}{SNR_{ab}})$ [25]. However, for a link with higher probability of disruption than *F_ab_*, it indicates that the environment of the acoustic channel here is rather harsh. In this case, in order to improve the restoration efficiency, we add the calculated position of the link to the periodic path of the glider until the environment there is improved. In our network model, we assume that the deployment of original sensor nodes has satisfied the coverage and connectivity conditions. Therefore, the probability of link disruption will not be high and that glider can complete its tasks of monitoring and restoring the link.

## 5. Performance Evaluation

In this section, we evaluate the performance of the glider-assisted link disruption restoration mechanism. To verify the effectiveness of the glider-assisted link disruption restoration mechanism proposed in this paper, experiments will be conducted from the aspects of packet delivery rate, end-to-end delay, connectivity and communication energy consumption of the network. The glider-assisted link disruption restoration mechanism is compared with the classic underwater routing protocols vector-based forwarding (VBF) [26], hop-by-hop vector-based forwarding (HHVBF) [27] and OVAR [13]. In VBF, a vector from the source to the sink is build and acts as the axis of the “routing pipe”. The data packet is transformed along the pipe. HH-VBF suggests the use of a routing vector for each individual forwarder in the network, instead of a single network-wide source-to-sink routing vector.

### 5.1. Experimental Setting

In the simulation, the water to be monitored in the simulation is a three-dimensional volume of 6 km × 4 km × 4 km. The initial deployment in the volume is composed of eight clusters, that is, the network contains eight clusters, and each cluster is responsible for monitoring the three-dimensional volume of 3 km × 2 km × 2 km. In each cluster there is a glider as an advanced node and common sensor nodes to carry out environmental monitoring tasks in their respective volumes. The initial energy, transmission power, receive power and communication distance of these common sensor nodes are all the same. The performance of the sensor carried on the underwater glider is not different from that of the common sensor nodes. The transmission power and receive power of the data are also consistent in every node. For source nodes, the data packet generation rate and sending rate follow the independent Poisson distribution process and the rate is λ (packets/s). 

The parameters used in the experiment refer to the Aqua-sent OFDM MODEM [28] and “Petrel” glider [11] parameters. The following simulation is done using Aqua-sim [29], which is an underwater network simulation software based on NS-2. The simulation parameters are listed in Table 1.

### 5.2. Evaluation with Different Parameters

Packet delivery rate (PDR) in the network refers to the ratio between the data packets (Ps) sent by the source node and the data packets (Pr) received by the target node, that is, the statistical measurement of correctly transmitting the data packet. It is an important performance indicator that reflects network reliability and communication quality. Therefore, we simulate the relationship of packet delivery rate and the different parameters *s*/*h* of four different algorithms, where *s* is the number of common sensor nodes in the network and *h* is the number of cluster heads in the network, that is, *s*/*h* is the number of common sensor nodes in a cluster.

**Figure 4:**
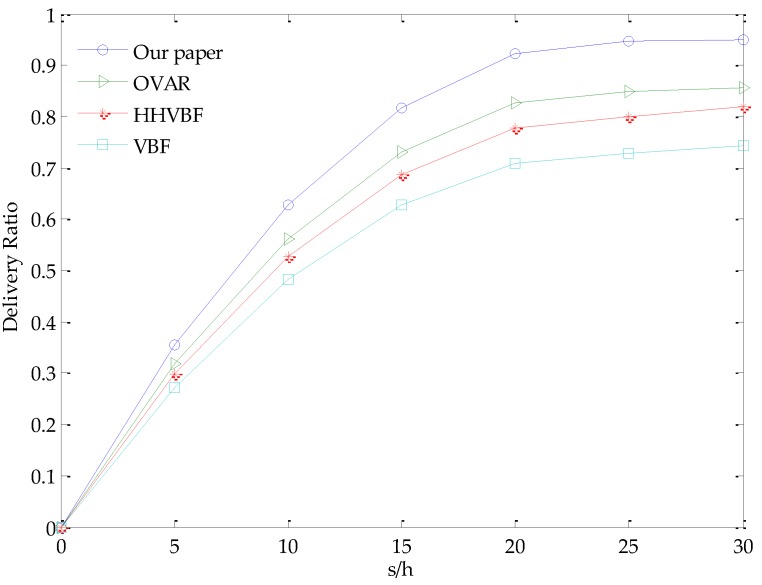
Relationship of the delivery ratio with different values of *s*/*h*.

In the simulation, the parameter *s*/*h* varies between 0 and 30 with a step of 5. As shown in Figure 4, by comparing the packet delivery rate with the change of the parameter *s*/*h*, we can conclude that the packet delivery rate is also closer to the ideal value 1 as the parameter *s*/*h* increases. The rate of increase of data packet delivery is faster at first and slower later because the network connectivity is particularly poor when the nodes are sparsely distributed. In this case, adding nodes will have a greater impact on network connectivity. However, when the number of nodes reaches a certain critical value, for example, when the parameter *s*/*h* in the figure is 20, the rate of packet delivery increases quite slowly because the network is basically connected. At this point if the number of nodes increases, the connectivity is improved less obviously. Therefore, in the following simulations, we perform other simulations based on the parameter *s*/*h* = 20. Compared with OVAR, HHVBF and VBF, the glider-assisted link-disruption restoration mechanism has a higher packet delivery rate with the same *s*/*h* value. For example, when the parameter *s*/*h* is 20, in the glider-assisted link-disruption restoration mechanism, the packet delivery rate has increased by 15%, 23% and 33%, respectively, relative to OVAR, HHVBF and VBF. This is because in the glider-assisted link disruption restoration mechanism, when a certain link in the network is disrupted due to change of underwater acoustic channel or external force in the ocean, the cluster head node in each sub-volume will notify the glider to perform restoring the link in time. For both the links among common nodes and the key nodes, the glider can do the repair, which greatly improves the delivery rate of the data packets in the network.

**Figure 5:**
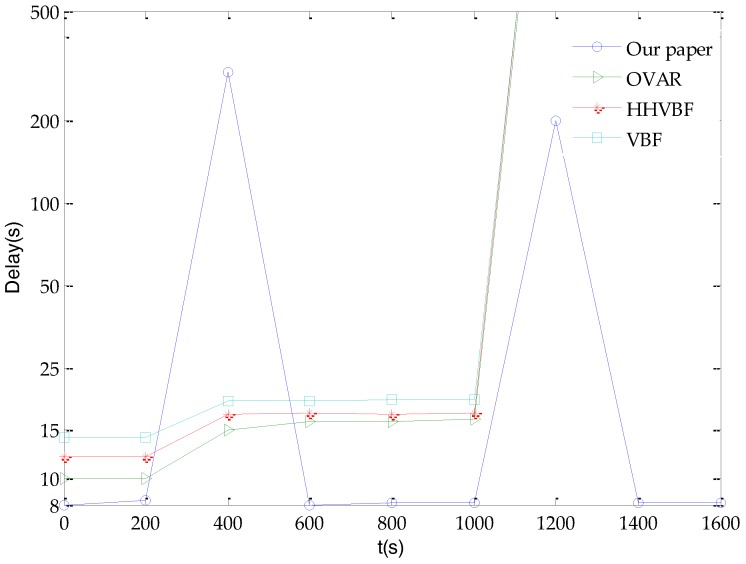
The end-to-end delay of network.

Based on the simulation in Figure 4, our simulation parameter *s*/*h* is 20. As shown in Figure 5, the end-to-end delay of the glider-assisted link-disruption restoration mechanism respectively is 20%, 38% and 43% lower than OVAR, HHVBF and VBF when there is no link disruption and the time is less than 200 s. This is because the glider-assisted link disruption restoration mechanism is based on the shortest path routing protocol. Shortest path routing has less time-delay than VBF and the HHVBF and OVAR protocols. For a sudden disruption of the link after 200 s, all of four algorithms restore the broken link. OVAR, HHVBF, and VBF rerouted the topology in a relatively shorter period of time by bypassing to repair the disrupted link. At this time, the glider-assisted link disruption restoration mechanism takes a relatively longer time to wait for the glider to assist in repairing the disrupted link. However, when the link returns to its original state, the glider-assisted link disruption restoration mechanism does not need to spend more time and energy on reconstructing the network topology to return to the original optimal state. OVAR, HHVBF, and VBF will continue to maintain the suboptimal state of the network for some time from 400 s to 1000 s in the following period, or spend a certain amount of time and energy on reconstructing back to the original optimal state. When a link in the network is disrupted for 1000 s, for the glider-assisted link disruption restoration mechanism, the previous process is continued for link restoration. For other algorithms, however, the link repair is not done within the simulation time. This may be due to the link between the key nodes being disrupted, for example, the link between the cluster head node and its upper-level node, so that the link cannot be repaired no matter how the path is detoured or the topology is reconstructed. Only when the link environment changes again these mechanisms may restore the disrupted link to the original state, thereby restoring link connectivity. At this time, the advantages of the glider-assisted link disruption restoration mechanism are obvious: the glider-assisted link disruption restoration mechanism has a strong general nature and it can adapt to a variety of different conditions.

**Figure 6:**
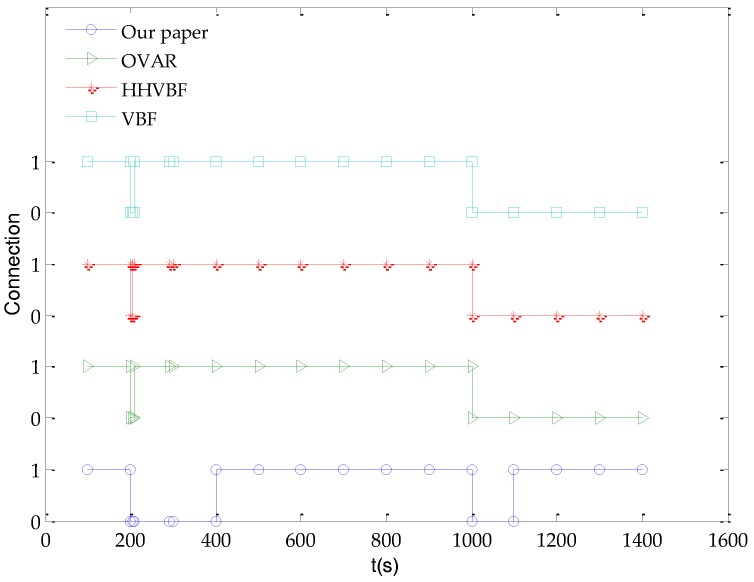
The connectivity of the network.

The scenario of Figure 6 is synchronized with Figure 5, which shows the connectivity of the link as time goes by. In this simulation, we use binary numbers to indicate the connectivity and disruption status of link. The state 1 indicates that the link is connected, and the opposite state 0 indicates the link is disrupted. As shown in Figure 6, when the link status changes from 1 to 0 at 200 s, the link is disrupted, and the OVAR, HHVBF and VBF resume the link connectivity within a relatively shorter period of time. The glider-assisted link disruption restoration mechanism takes a relatively longer time to complete the link restoration task, and the link status changes from 0 to 1, until 1000 s, the link state has changed from 1 to 0, that is, another link disrupted. However, the OVAR, HHVBF and VBF cannot complete the link repairing during the simulation time. However, the glider-assisted link disruption restoration mechanism still completes the link restoration without any difference and restores the initial connectivity of the network. This shows that the glider-assisted link disruption restoration mechanism is more generally applicable.

**Figure 7:**
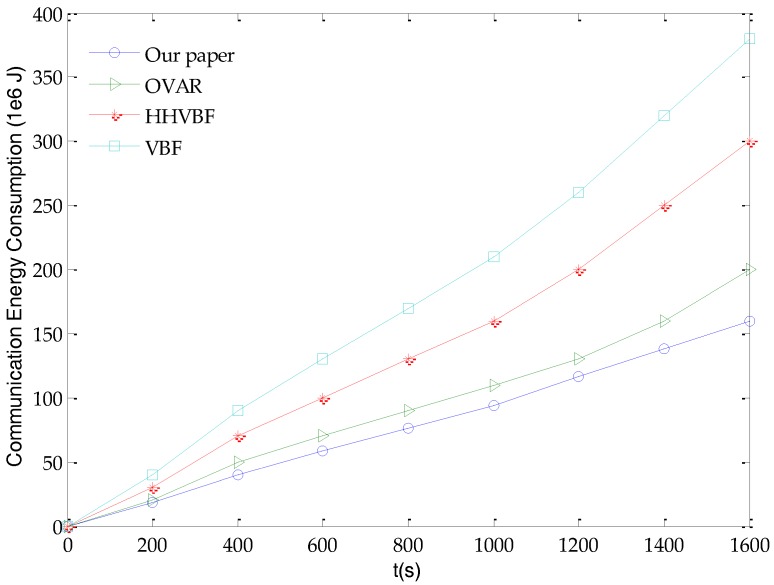
The change of communication energy consumption over time.

Energy is also an important factor to consider since the common sensor nodes in UASNs are mostly battery-powered. The storage capacity of batteries is limited and underwater charging technology is more difficult to achieve. For common nodes, its energy consumption is mainly used to collect sensor data, send data packets, forward data packets and receive data packets, and we define these energy consumption as communication energy consumption. Therefore, communication energy consumption is an important measure of the life of a common sensor node. That is, the less energy consumption to deliver the same packet to the aggregation node can prolong the life of the node, thereby prolonging the life of the entire network. Therefore, in order to verify the performance of the glider-assisted link-disruption restoration mechanism, we compare its communication energy consumption with OVAR, HHVBF and VBF, as shown in Figure 7.

It can be seen from Figure 7 that the increase rate of communication energy consumption of the glider-assisted link disruption restoration mechanism is almost unchanged as the rate of increase of OVAR, HHVBF and VBF increases. The reason is that when performing link restoration, the OVAR, HHVBF and VBF algorithms need to perform more data packet retransmissions to select the more appropriate link. The process of topology reconstruction at this time consumes more energy. The glider-assisted link disruption restoration mechanism just added a node, so the topology is not changed much, so there is no extra energy costs. Therefore, the increase rate of the glider-assisted link disruption restoration mechanism is stable regardless of whether a disrupted link is restored. As shown in Figure 7, the glider-assisted link-disruption restoration mechanism can consume less energy than the OVAR, HHVBF, and VBF algorithms, respectively. For example, the communication energy consumption is reduced by 20%, 47% and 58%, respectively, at 1600 s. This proves that the glider-assisted link-disruption restoration mechanism has obvious advantages in reducing communication energy consumption.

## 6. Conclusions

In this paper, we design a glider-assisted linkdisruption restoration mechanism for UWANs, which includes a LDR-GS mechanism and GALO. In LDR-GS, when any cluster head node discovers a link disruption among cluster members, the corresponding link state flag in every cluster head node becomes 1. On the other hand, a glider periodically approaches the cluster head node and the corresponding link state flag in the gliders also becomes 1. Then the glider is scheduled to go to the location for restoring a link disruption, but due to the limited trajectory of the underwater glider, we further design GALO to improve the efficiency of the glider-assisted link disruption restoration mechanism. In GALO, we combine the specific sawtooth movement of the glider and the channel attenuation model to select the optimal relay location. This can enable underwater gliders to accurately restore network connectivity as relay nodes of the disrupted links. We evaluate the performance of different parameters on the glider-assisted link disruption restoration mechanism and compare it with OVAR, HHVBF and VBF algorithms. Simulation results show that the communication energy consumption is reduced by about 20~58% compared with other algorithms and the packet delivery rate is also increased about 15~33% because the glider-assisted link disruption restoration mechanism can change the topology of the entire network to a lesser degree during execution. Compared with other algorithms, the glider-assisted link disruption restoration mechanism proposed can flexibly complete restoring disrupted links because it can schedule free-moving gliders for link restoration. For any link, it can complete the restoring task, while other algorithms have limitations for some link disruptions among key nodes. Therefore, the glider-assisted link disruption restoration mechanism is more general for restoring link disruptions and reinforcing the connectivity in UASNs.

## Figures and Tables

**Table 1:** Simulation parameters.

Name	Values
Monitoring volume	6 km × 4 km × 4 km
Transmission speed	1.5 km/s
Transmission range	1.5 km
DATA packet size	300 B
Maximum gliding depth	4 km
steady-state gliding maximum pitch angle	69.5°
steady-state gliding minimum pitch angle	9.2°
Maximum gliding speed	2 Kn (1.852 km/h)

## References

[1] Peng J., Xu Y., Liu J. (2017). A Distributed and Energy-Efficient Algorithm for Event K-Coverage in Underwater Sensor Networks. Sensors.

[2] Akyildiz I.F., Pompili D., Melodia T. (2005). Underwater acoustic sensor networks: Research challenges. Ad Hoc Netw..

[3] Taehee Won S.P. (2012). Design and Implementation of an Omni-Directional Underwater Acoustic Micro-Modem Based on a Low-Power Micro-Controller Unit. Sensors.

[4] Lloret J. (2013). Underwater sensor nodes and networks. Sensors.

[5] Sendra S., Lloret J., Jimenez J.M., Parra L. (2016). Underwater Acoustic Modems. IEEE Sens. J..

[6] Latif K., Javaid N., Ahmad A., Khan Z.A., Alrajeh N., Khan M.I. (2016). On Energy Hole and Coverage Hole Avoidance in Underwater Wireless Sensor Networks. IEEE Sens. J..

[7] Nguyen S.T., Cayirci E., Yan L., Rong C. (2009). A shadow zone aware routing protocol for acoustic underwater sensor networks. IEEE Commun. Lett..

[8] Mahboubi H., Vaezi M., Labeau F. (2017). Mobile Sensors Deployment Subject to Location Estimation Error. IEEE Trans. Veh. Technol..

[9] Chen B., Pompili D. (2015). Modeling position uncertainty of networked autonomous underwater vehicles. Ad Hoc Netw..

[10] Liu F., Wang Y., Niu W., Ma Z., Liu Y. Hydrodynamic performance analysis and experiments of a hybrid underwater glider with different layout of wings. Proceedings of the Oceans 2014.

[11] Liu F., Wang Y., Wang S. (2014). Development of the Hybrid Underwater Glider PetreI-II. Sea Technol..

[12] Sheng M., Li X., Wang X., Xu C. (2017). Topology Control with Successive Interference Cancellation in Cognitive Radio Networks. IEEE Trans. Commun..

[13] Ghoreyshi S.M., Shahrabi A., Boutaleb T. (2016). A Novel Cooperative Opportunistic Routing Scheme for Underwater Sensor Networks. Sensors.

[14] Zhang Y., Chen W., Liang J., Zheng B., Jiang S. (2015). A Network Topology Control and Identity Authentication Protocol with Support for Movable Sensor Nodes. Sensors.

[15] Jin Z., Ma Y., Su Y., Li S., Fu X. (2017). A Q-Learning-Based Delay-Aware Routing Algorithm to Extend the Lifetime of Underwater Sensor Networks. Sensors.

[16] Santos R., Orozco J., Micheletto M., Ochoa S.F., Meseguer R., Millan P., Molina A.C. (2017). Real-Time Communication Support for Underwater Acoustic Sensor Networks. Sensors.

[17] Guo Y.B., Guo Y.B., Zhan Y.Z. (2010). Security Topology Control Method for Wireless Sensor Networks with Node-Failure Tolerance Based on Self-Regeneration.

[18] Carmen D.M. (2009). A Topology Reorganization Scheme for Reliable Communication in Underwater Wireless Sensor Networks Affected by Shadow Zones. Sensors.

[19] Dong C., Guo L., Yin J. Coverage control study of mobile uwasns nodes based on particle swarm optimization algorithm. Proceedings of the 11th ACM International Conference on Underwater Networks & Systems.

[20] Pandey P., Hajimirsadeghi M., Pompili D. (2014). Region of Feasibility of Interference Alignment in Underwater Sensor Networks. IEEE J. Ocean. Eng..

[21] Ibrahim S., Al-Bzoor M., Liu J., Ammar R., Rajasekaran S., Cui J.H. (2013). General optimization framework for surface gateway deployment problem in underwater sensor networks. EURASIP J. Wirel. Commun. Netw..

[22] Pompili D., Melodia T., Akyildiz I.F. (2009). Three-dimensional and two-dimensional deployment analysis for underwater acoustic sensor networks. Ad Hoc Netw..

[23] Liu J., Wang Z., Peng Z., Cui J.H. Suave: Swarm underwater autonomous vehicle localization. Proceedings of the IEEE INFOCOM 2014—IEEE Conference on Computer Communications.

[24] Yoon S., Azad A.K., Oh H., Kim S. (2012). AURP: An AUV-Aided Underwater Routing Protocol for Underwater Acoustic Sensor Networks. Sensors.

[25] Liu Z., Guan Q., Chen F., Liu Y. Outage probability analysis for unmanned underwater vehicle based relaying. Proceedings of the 11th ACM International Conference on Underwater Networks & Systems.

[26] Xie P., Cui J.H., Lao L. VBF: Vector-Based Forwarding Protocol for Underwater Sensor Networks. Proceedings of the 5th international IFIP-TC6 conference on Networking Technologies, Services, and Protocols; Performance of Computer and Communication Networks; Mobile and Wireless Communications Systems.

[27] Nicolaou N., See A., Xie P., Cui J.H., Maggiorini D. Improving the Robustness of Location-Based Routing for Underwater Sensor Networks. Proceedings of the Oceans 2007.

[28] Yan H., Zhou S., Shi Z.J., Li B. A DSP implementation of OFDM acoustic modem. Proceedings of the Workshop on Underwater Networks.

[29] Xie P., Zhou Z., Peng Z., Yan H. Aqua-Sim: An NS-2 based simulator for underwater sensor networks. Proceedings of the MTS/IEEE Biloxi—Marine Technology for Our Future: Global and Local Challenges (OCEANS 2009).

